# S6-5 Tool for supporting implementation of physical activity programs among older adults: from science to practice

**DOI:** 10.1093/eurpub/ckad133.032

**Published:** 2023-09-11

**Authors:** Liesbeth Preller

**Affiliations:** Dutch Knowledge Centre for Sport & Physical Activity

## Abstract

**Aim:**

Research shows that professionals in healthcare and social work are crucial for reaching older adults but often do not refer them to local PA programs. Multidisciplinary collaboration, including sports professionals, is challenging. In The Netherlands interlocal differences in collaboration are large due to influence of local policy. Our aim was to develop a tool which makes referral of older clients to PA groups efficient and effective.

**Methods:**

For input on contents of the tool we used a sounding board group, with representatives of three groups of professionals: physician assistant of the general practitioner, geriatrics physical therapist, and neighbourhood sports coach (NSC; connecting sports sector with other sectors like healthcare). During meetings we discussed which type and contents would help to improve collaboration and referral. We subsequently built an online tool and asked feedback on design and contents.

**Result:**

The professionals stated that not knowing each other, unfamiliarity with each other’s role and with suitable local PA programs, are barriers for referring to PA programs and facilities. The developed generic tool describes referral procedures for four different groups of community-dwelling older people, ranging from vital to vulnerable. Basically one starts with screening on quantitative PA behaviour, motives and barriers, and leads the person directly or through an intermediate professional to suitable PA. In addition it includes general information on health effects of PA, screening methods, general PA possibilities and roles of different professionals in the referral process. For use in the local setting the tool has to be tailored, generally by the NSC, but with involvement of local organisations. Tailoring means that contact information of local professionals and organisations, local PA groups, and specific local agreements on collaboration are added.

**Conclusion:**

Healthcare and social workers generally don’t refer older inactive older adults to suitable PA groups because they lack insight into local PA programs, and don’t know the relevant professionals. Since the tool makes this information visible at the local level, this will lower the threshold to refer clients adequately to PA.

